# Why acute decreased vision in patients with high myopia is an emergency?

**DOI:** 10.11604/pamj.2014.19.281.5528

**Published:** 2014-11-14

**Authors:** Hajji Chaimae, Rajae Daoudi

**Affiliations:** 1 Université Mohammed V Souissi, Service d'Ophtalmologie A de l'Hôpital des Spécialités, Centre Hospitalier Universitaire, Rabat, Maroc

**Keywords:** High myopia, choroidal neovascularization, eye, anti-vascular endothelial growth

## Image in medicine

We report a case of 43 years old women who present myopic CNV (A) causing an acute vision loss. That has not been treated at time causing a chorioretinal atrophy as the angiography (B) and her Optical Coherence Tomography (OCT) (C) shows. High myopia is a major cause of legal blindness in many countries, especially in young people. Itis defined as a refraction of at least -6.00 diopters and/or an axial length upper than 25.5 mm. Excessive axial elongation of the globe in high myopia can cause mechanical stretching of the choroid and retinal layers, causing a degenerative changes. It is well known that individuals with high myopia have increased risks of retinal complications such as peripheral retinal degenerations, retinal detachment, choroidal neovascularisation (CNV) and macular haemorrhage. Among various lesions associated with high myopia, macular CNV is the most vision threatening complications. It develops in around 10% of eyes with high myopia. CNV is the creation of new blood vessels in the choroid layer of the eye. The disease can progress to chorioretinal atrophy (Fuchs spot). Patients may develop metamorphopsia, central scotoma and decreased visual acuity. Recently, the use of angiogenesis therapy with anti-vascular endothelial growth factor (VEGF) agents like intravitreal bevacizumab has demonstratedencouraging results and hadbecome the treatment of choice for myopic CNV.

**Figure 1:**
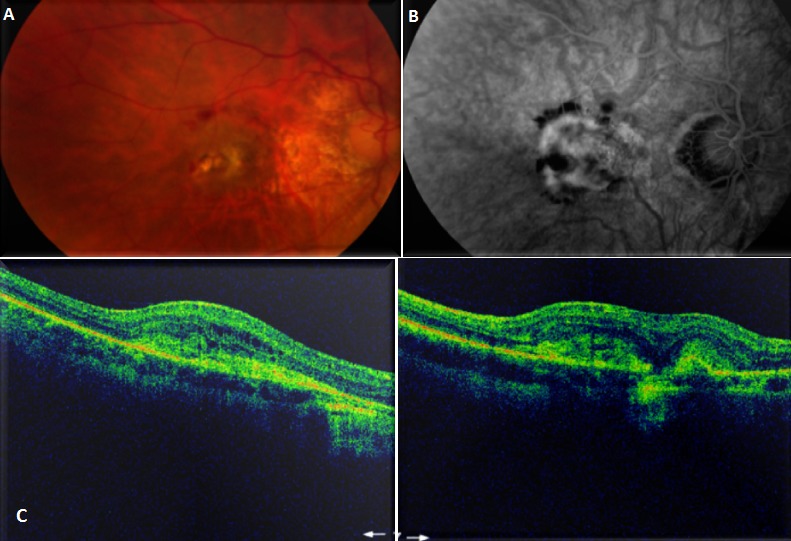
Myopic CNV (A) causing an acute vision loss. That has not been treated at time causing a chorioretinal atrophy as the angiography (B) and the her Optical Coherence Tomography (OCT) (C) shows

